# An update review of the application of single-cell RNA sequencing in pregnancy-related diseases

**DOI:** 10.3389/fendo.2024.1415173

**Published:** 2024-12-09

**Authors:** Zhiyi Zhou, Xiuhua Yang

**Affiliations:** Department of Obstetrics, The First Hospital of China Medical University, Shenyang, China

**Keywords:** pregnancy, placenta, scRNA-seq, recurrent pregnancy loss, preeclampsia, preterm birth, gestational diabetes mellitus

## Abstract

Reproductive success hinges on the presence of a robust and functional placenta. Examining the placenta provides insight about the progression of pregnancy and valuable information about the normal developmental trajectory of the fetus. The current limitations of using bulk RNA-sequencing (RNA-seq) analysis stem from the diverse composition of the placenta, hindering a comprehensive description of how distinct trophoblast cell expression patterns contribute to the establishment and sustenance of a successful pregnancy. At present, the transcriptional landscape of intricate tissues increasingly relies on single-cell RNA sequencing (scRNA-seq). A few investigations have utilized scRNA-seq technology to examine the codes governing transcriptome regulation in cells at the maternal-fetal interface. In this review, we explore the fundamental principles of scRNA-seq technology, offering the latest overview of human placental studies utilizing this method across various gestational weeks in both normal pregnancies and pregnancy-related diseases, including recurrent pregnancy loss (RPL), preeclampsia (PE), preterm birth, and gestational diabetes mellitus (GDM). Furthermore, we discuss the limitations and future perspectives of scRNA-seq technology within the realm of reproduction. It seems that scRNA-seq stands out as one of the crucial tools for studying the etiology of pregnancy complications. The future direction of scRNA-seq applications may involve devolving into functional biology, with a primary focus on understanding variations in transcriptional activity among highly specific cell populations. Our goal is to provide obstetricians with an updated understanding of scRNA-seq technology related to pregnancy complications, providing comprehensive understandings to aid in the diagnosis and treatment of these conditions, ultimately improving maternal and fetal prognosis.

## 1 Introduction

Reproductive success hinges on the presence of a robust and functional placenta. The placenta is widely acknowledged as the central organ for facilitating nutrient transfer, maintaining immune tolerance, and enabling pregnancy adaptation between the mother and the offspring. The development of the placenta involves a sequence of crucial components, including the differentiation of extra-embryonic cells, the growth of blood vessels, and uterine artery remodeling (1, 2). The placenta comprises three primary types of epithelial trophoblasts: villi cytotrophoblasts (VCTs), syncytiotrophoblasts (STBs), and extravillous trophoblasts (EVTs) (3). The layer of multinucleated STBs situated above VCTs directly interfaces with maternal blood. The structure of placental villi also includes fetal macrophages, known as Hofbauer cells, and fetal capillaries to ensure adequate blood perfusion throughout the placenta. A specific category of trophoblast cells, called EVTs, invades decidual tissues to remodel maternal spiral arteries and interacts with different lymphocytes to prevent the allo-rejection of the fetus. Although the classification of cells in the placenta has been sorted following the aforementioned description, there is still uncertainty regarding the usefulness of defining subcategories for trophoblast and stromal cells. Moreover, the relationships and functions between these cell subtypes remain indistinct.

Examining the placenta provides insight about the progression of pregnancy and valuable information about the normal developmental trajectory of the fetus. To gain a deeper understanding of the mechanisms underlying healthy pregnancies and severe pregnancy complications, several studies have employed microarrays and RNA-sequencing techniques to analyze gene expression patterns in the placenta (4). However, the current limitations of using bulk RNA-sequencing (RNA-seq) analysis stem from the diverse composition of the placenta, hindering a comprehensive description of how distinct trophoblast cell expression patterns contribute to the establishment and sustenance of a successful pregnancy. At present, the transcriptional landscape of intricate tissues increasingly relies on single-cell RNA sequencing (scRNA-seq) (5). The enhanced resolution provided by scRNA-seq enables the detection of rare cell types that may remain unidentified in tissue-level transcriptomes due to signal dilution (6–8). Several studies have demonstrated that scRNA-seq has the ability to capture various cell types present in the placenta and distinguish their origin as either maternal or fetal source (1, 9, 10). Furthermore, it has been observed that placental signatures derived from single cells can be detected in maternal circulating cell-free RNA (10), indicating the possibility of non-invasively identifying pregnant women who may develop early-onset PE (EOPE). Recently, a few investigations have utilized scRNA-seq technology to examine the codes governing transcriptome regulation in cells at the utero-placental interface (9, 11). These studies have unveiled the distribution and developmental trajectory of cell subtypes within the decidua, as well as elucidated how immune tolerance is regulated during the process of uterine spiral artery remodeling in pregnancy.

In this review, we explore the fundamental principles of scRNA-seq technology, offering the latest overview of human placental studies utilizing this method across various gestational weeks in both normal pregnancies and pregnancy complications, including recurrent pregnancy loss (RPL), preeclampsia (PE), preterm birth, and gestational diabetes mellitus (GDM). Additionally, we introduce various computational analysis platforms and resources accessible to the general population. Furthermore, we discuss the limitations and future perspectives of scRNA-seq technology within the realm of reproduction. Undoubtedly, scRNA-seq studies related to pregnancy in other mammals have provided a novel perspective on human reproductive safety across different species (12). However, due to the scope of this manuscript, we will not delve into animal assays. Our goal is to provide obstetricians with an updated understanding of scRNA-seq technology related to pregnancy complications, providing comprehensive understandings to aid in the diagnosis and treatment of these conditions, ultimately improving maternal and fetal prognosis.

## 2 scRNA-seq technology

Since the introduction of the initial scRNA-seq technique in 2009 (13), this method has gained extensive popularity for investigating gene expression dynamics at an individual cell level across various tissues. This approach differs significantly from typical bulk RNA-seq techniques that examine the transcriptome profile of an entire cell population. On the contrary, scRNA-seq enables the examination of gene expression with significantly enhanced precision: at the individual cellular level. Since its inception, scRNA-seq has evolved from analyzing ten cells to analyzing tens of thousands of cells in a single analysis. With the development of science, scRNA-seq technology has gradually become faster and cheaper (14). The utilization of scRNA-seq enables the identification and analysis of cell types existing in the specimen, along with their patterns of gene expression. Additionally, scRNA-seq offers the biology of various cell types and their interactions in both healthy and diseased conditions. This approach also allows for the characterization of cells exhibiting atypical expression profiles (outlier cells), thereby offering potential novel understandings into the mechanisms underlying disease progression (15).

**Figure 1:**
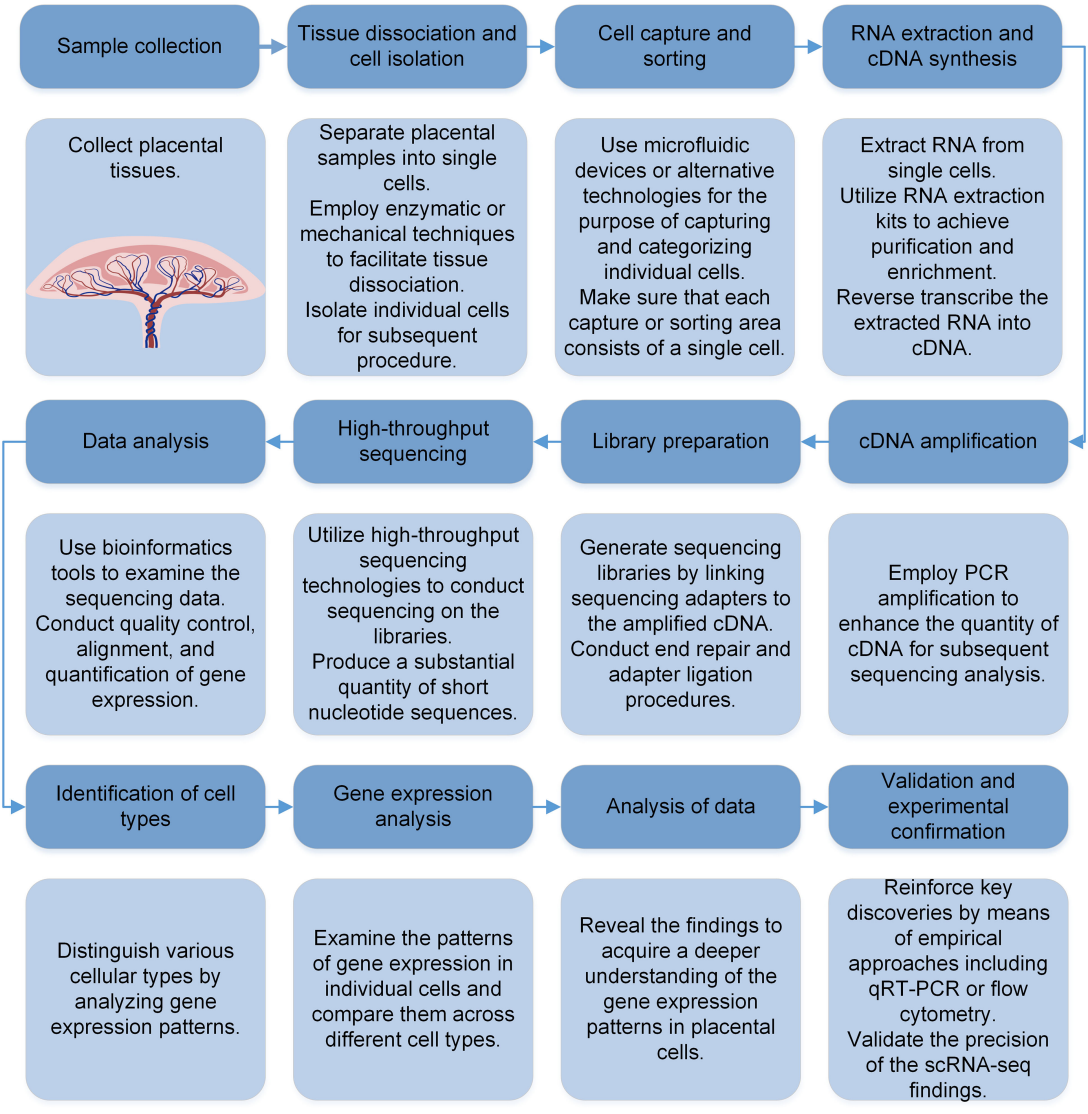
The workflow of scRNA-seq technique in placental cells. cDNA, complementary DNA.

The typical procedure for scRNA-seq in placenta involves the isolation of individual cells, the preparation of scRNA-seq libraries, sequencing, and computational analysis (**Figure 1**). In placental or decidual studies, the technology of scRNA-seq is commonly utilized and easily accessible across several platforms (e.g. Fluidigm C1, Drop-seq, 10x Genomics Chromium Next GEM Single Cell 50/30, Takara Bio SMART-seq, BD Rhapsody, NovaSeq, Illumina HiSeq, Python) (14). Library preparation methods include Smart-seq2 (16), as well as other methods combined with unique molecular identifiers (UMI) or barcodes (17, 18). Generally, bioinformatics analysis pipelines consist of techniques related to the following issues: batch-adjustment, standardization and ensuring data quality, selecting features and reducing dimensionality, clustering without supervision and annotating cell types with guidance, analysis of differential expression, recreating timing patterns, or analyzing the path of movement, and analysis of intercellular communication. To obtain single cells for analysis, cells from either cultured or tissue samples undergo enzymatic or mechanical disruption and then pass through cell strainer. These strainers trap cell clusters while allowing individual cells to pass through. Subsequently, the collected cells are rinsed to eliminate extraneous RNA from disrupted cells. Further enrichment is possible through gradient centrifugation or flow cytometry into individual wells. On the other hand, microfluidic systems are employed to load single-cell suspensions, allowing only one cell to pass through and be trapped within a gel emulsion matrix (GEM). Each GEM, or well, is equipped with a distinct cellular barcode and contains all the essential reagents for reverse transcription and the preparation of next-generation sequencing libraries (19). By sequencing and receiving millions of reads, each carrying a unique cell barcode, cDNA insert, and unique molecule identifiers, it becomes feasible to determine mRNA copy numbers.

Despite the advancements in experimental technologies and analytical methods, it is important to acknowledge the obstacles associated with the widespread adoption of scRNA-seq. For instance, the process of scRNA-seq involves the mechanical or enzymatic separation of the sample, which may lead to the exclusion of susceptible cell types or potentially modify gene expression within cells. Therefore, it is crucial to fine-tune dissociation procedures according to the particular tissue types under investigation to minimize any potential bias introduced during dissociation. Moreover, it is essential to conduct a thorough evaluation of the single cell preparation to verify the viability and complete dissociation of cells into individual entities before their introduction onto the microfluidic chip. Two scRNA-seq studies have indicated that the frequencies of specific cell types in the placenta could be influenced by tissue dissociation (11, 20). Variations in outcomes can occur due to both the experimental and data analysis stages. In addition, it is essential to confirm the findings of scRNA-seq by employing supplementary techniques like immunofluorescence, flow cytometry, or RNAscope. Genetic perturbations, such as the manipulation of the specific gene through knockdown, knockout, or overexpression techniques, should be performed to investigate the biological importance of these various sub-populations. There exist multiple tactics, methodologies, and instruments for conducting the analysis of scRNA-seq data. Therefore, when selecting approaches for a specific investigation, it is crucial to consider factors such as the study design, the type of scRNA-seq technology employed, and the outcomes obtained from extensive evaluations of alternative methods for each analysis stage.

## 3 Normal pregnancy

A series of scRNA-seq studies were conducted on human placental tissues at various gestational stages during a normal pregnancy (**Table 1**). These relevant scRNA-seq investigations have successfully unveiled novel cellular subcategories and anticipated intercellular associations by scrutinizing the variability in gene expression among cells. In one particular study, the gene expression profile of over 70,000 cells derived from the feto-maternal interface were examined from 6 to 14 gestational weeks (9). They found that placental samples predominantly consisted of fetal cells, with a minor presence of maternal macrophages. Conversely, in decidual samples, the majority of cells, except for a small number of EVTs, originated from the mother (9). Through unsupervised clustering and analysis of differential gene expression, two groups of decidual fibroblast cells, three sets of decidual stromal cells, as well as three subsets of decidual natural killer (dNK) cells were established (9). Furthermore, a statistical tool called CellPhone was developed to detect precise connections between dNK cells and infiltrating fetal EVTs, as well as maternal immune and stromal cells. By utilizing this tool, predictions were made about potential ligand-receptor interactions that could regulate the differentiation of trophoblast cells into STBs or EVTs (9). Another team conducted scRNA-seq analysis on villous cells at 8 weeks and decidual cells at 24 weeks obtained from human placental tissues, and identified a total of 14 unique subtypes of placental cells (20). In their examination of the transcriptome in the villous region, researchers identified three distinct subtypes of cytotrophoblasts (CTBs), two subtypes of EVTs, two subtypes of mesenchymal stromal cells expressing CD90, ENG, and CD74 simultaneously, two subtypes of Hofbauer cells positive for CD68, as well as a population of STB (20). Suryawanshi and colleagues conducted scRNA-seq on 8 villous samples and 8 matched maternal decidual tissues obtained during 6-11 weeks of gestation (11). Utilizing data from villous samples, they characterized different trophoblast subtypes, such as CTB, EVT, and STB. Furthermore, various cell populations within the villous cores, including Hofbauer cells, endothelial cells, and mesenchymal stromal cells, were identified. Another study employed scRNA-seq to analyze a total of 50,790 cells (21). This research included 7,798 cells obtained from healthy placental villi from 7 pregnant females in early pregnancy, as well as an additional 6,309 cells derived from trophoblast organoid cultures. They observed various trophoblast states, such as precursor CTB, column CTB, STB precursors, and EVT. The reconstruction of the lineage trajectory revealed a common ancestral source replicated in organoids derived from human trophoblast stem cells. This study provides a detailed analysis of the diverse characteristics of trophoblasts in early pregnancy and successfully uncovers gene networks in human CTB progenitors. It also highlights the significance of basal cell adhesion molecule (BCAM) as a potential marker and regulator for primitive progenitor cells (21). Another study focused on the gene expression patterns of placental cells at full term (1). It conducted a comparison between the transcriptomes of individual cells and the transcriptome data at the tissue level. The results demonstrated that scRNA-seq analysis successfully identified over 4,000 genes not detected in the transcriptome data obtained from tissues, supporting the notion that scRNA-seq has the potential to unveil expression profiles of uncommon cell types.

**Table 1:** Human placental scRNA-seq studies in a normal pregnancy.

Reference	Gestational age (weeks)	Population	Samples	Number of cells	Single cell isolation	Platform	Average reads length (bp)	Mean pair-end reads	Data repository
Pavlicev (1)	Term pregnancy	American	Two placentas from C-section without labor	87	Enzyme, gradient centrifugation	Fluidig C1 and Laser microdissection	75	3.65 × 10^6^	GEO: GSE87726
Vento-Tormo R (9)	6-14 weeks	British	11 deciduae and 5 placentas	70,000	Enzyme, gradient centrifugation, FACS	10× Genomics v2, Smart-seq2	75	1 × 10^6^	ArrayExpress: E-MTAB-6701, E-MTAB-6678, and E-MTAB-7304
Liu (20)	8 weeks and 24 weeks	Chinese	8 placentas	1,471	Enzyme, gradient centrifugation, MACS	Smart-seq2	150	1 × 10^6^	GEO: GSE89497
Suryawanshi (11)	6-11 weeks	American	8 villi and 6 deciduae	14,341, 6,754	Enzyme	10× Genomics and Drop-seq	101	N/A	BioProject: PRJNA492324
Sun (65)	11-13 weeks	American	10 placental tissues	7,245	Enzyme	10× Genomics	2 × 75	1.901 ×10^7^	GEO: GSE131696
Pique-Regi (60)	The second and third trimesters	American	32 placental villi or decidua samples	26,501	Enzyme, gradient centrifugation	10× Genomics 3’	N/A	N/A	NIH dbGAP: phs001886.v2. p1
Shannon (21)	5-12 weeks	Canadian	9 placental tissues	50,790	N/A	10× Genomics	50,000	N/A	ArrayExpress: E-MTAB-6701
Wang (85)	Full-term pregnancy	Chinese	8 placentas	11,438	N/A	10× Genomics	N/A	N/A	CNSA: CNP0000878
Li (86)	6-16 weeks	Chinese	11 placentas	52,179	N/A	10× Genomics	N/A	150	GSA: HRA003309

FACS, fluorescence-activated cell sorting; MACS, magnetic activated cell sorting; N/A, not available.

Aside from the placenta, single-cell techniques are currently being utilized to analyze various biological specimens from pregnant women. Researchers have developed a comprehensive map of individual cells in the human myometrium, uncovering alterations in intercellular communication during the natural process of term labor (22). The primary finding of this research indicates that both non-immune and immune cells play a role in the contraction and inflammatory response during the labor process at full term (22). This study further suggests that the maternal whole-blood transcriptome during pregnancy can be employed to quantify single-cell signatures derived from the myometrium (22). These signatures are found in higher abundance among women experiencing labor, suggesting a potential noninvasive approach for monitoring pregnancy and its associated complications.

The analysis of maternal peripheral blood is essential for understanding the immune mechanisms and biomarkers associated with placental development. A pivotal study has led the way in creating a comprehensive atlas of maternal peripheral blood mononuclear cell (PBMC) through scRNA-seq. This study specifically focused on exploring immune adaptations during pregnancy (23). This report established a theoretical foundation for understanding the maternal-fetal immune mechanism and the pathogenesis of adverse pregnancy outcomes.

Research on umbilical cord blood plays a crucial role in understanding the progression of normal pregnancy. Recent findings have unveiled diverse cellular populations within umbilical cord blood, including erythroid cells, erythroid precursor cells, six subtypes of erythroid cells, T cells, B cells, NK cells, and endothelial progenitor cells (24). The revelation of these findings is poised to enhance the efficacy of cord blood stem cell transplantation by identifying specific subgroups or manipulating their genetic expression. Furthermore, an investigation into immune cells in the umbilical cord blood of newborns has identified variations in gene expression among T and B cell subtypes, setting them apart from their adult counterparts (25). This study contributes to a deeper understanding of the immune tolerance exhibited by newborns.

Recently, scRNA-seq technology has been used to study the effects of adverse environmental exposures (such as alcohol and electronic cigarettes) on reproduction (26). One research utilized scRNA-seq to examine the effects of fine particulate matter (PM2.5) on uterine cell populations and gene expression patterns in mice in estrus and the first trimester of pregnancy (27). A significant reduction in the percentage of NK cells was noted in mice exposed to PM2.5. Additionally, it revealed that the IL-17 signaling pathway in NK cells was suppressed, indicating it as a primary mechanism of PM2.5-induced toxicity (27). Furthermore, scRNA-seq was utilized to study the mechanisms behind age-associated male fertility (28). Testicular biopsies were obtained from three young men and three old men (28). It was discovered that impaired DNA repair in spermatogonial stem cells (SSCs) could potentially contribute to the accumulation of new germline mutations as age increases. Age-related decline in redox balance was observed in aged Leydig cells (LCs), and the use of antioxidant drugs improved cellular function and enhanced testosterone synthesis in LCs (28). These results offer a thorough comprehension of the specific cellular processes responsible for human testicular aging at a single-cell level, and propose possible treatment targets to combat age-related decline in male fertility and hypogonadism.

## 4 RPL

RPL, which refers to the occurrence of two or more consecutive pregnancy losses, impacts approximately 5% of women attempting to achieve conception (29, 30). Potential factors leading to RPL may include genetic abnormalities, endocrine imbalances, uterine structural anomalies, as well as other significant elements such as thrombophilia and maternal infections (31). The cause of RPL remains unidentified in approximately half of the cases. Several reports have provided a comprehensive analysis of the cellular makeup and interactions within the maternal-fetal interface in RPL patients by using scRNA-seq technologies, offering detailed information about the diverse decidual cell types, their functions, and communication mechanisms (**Table 2**).

**Table 2:** Human scRNA-seq studies at maternal-fetal interface in recurrent pregnancy loss (RPL).

Reference	Gestational age (weeks)	Population	Samples	Number of cells	Single cell isolation	Platform	Average reads length (bp)	Mean pair-end reads	Data repository
Lucas (36)	Peri-implantation	British	Endometrium from 3 RPL and 3 normal pregnancies	4,580	N/A	Drop-Seq	N/A	N/A	GEO: GSE127918
Du (32)	5-8 weeks	Chinese	Deciduae from 6 RPL and 5 normal pregnancies	66,078	N/A	10× Genomics	N/A	150	SRA: PRJNA672658
Guo (33)	7-9 weeks	Chinese	Decidual samples from 9 RPL and 15 normal pregnancies	18,646	Enzyme, gradient centrifugation	10× Genomics	N/A	150	GSA: CRA002181
Chen (34)	6-9 weeks	Chinese	Deciduae from 3 RPL and 3 normal pregnancies	13,953	Enzyme, gentleMACS, centrifugation	10× Genomics	N/A	N/A	GEO: GSE164449
Wang (37)	6-8 weeks	Chinese	Deciduae from 3 RPL and 3 normal pregnancies	56,758	Enzyme, centrifugation	10× Genomics	98	40,000	GSA: HRA000237 with BioProject: PRJCA003061
Pan (35)	RPLgrou: 7.66 weeks; Control group: 7.24 weeks	Chinese	The maternal-fetal interface samples from 3 RPL and 3 normal pregnancies	63,249	Enzyme	NovaSeq	N/A	150	N/A
Zhu (87)	6-12 weeks	Chinese	Decidual tissues from 46 controls and 31 RPL patients	19,416	Enzyme, centrifugation	Illumina HiSeq X Ten	N/A	N/A	N/A

A study was conducted involving a combined count of 66,078 individual cells derived from decidual samples obtained from 6 patients experiencing RPL and 5 females without any health issues serving as controls (32). As revealed by their scRNA-seq findings, the RPL samples exhibited disrupted decidualization and significantly impaired intercellular communication between stromal cells and other cellular populations, including aberrant stimulation of macrophage cells and NK cells (32). Another research effort investigated RPL by conducting scRNA-seq on 24 decidual samples obtained during the first trimester (33). These samples were preselected for CD45^+^ cells, with 9 samples from individuals experiencing RPL and 15 samples from healthy pregnant women. RPL patients showed a reduction in the percentage of dNK cells that promote embryo development, which can be attributed to an atypical development of NK cells in RPL (33). Additionally, they found that the connections between immune cell subtypes and EVTs are disturbed in RPL patients (33). Another study examined decidual transcriptomes of 13,953 CD45^+^ cells derived from three healthy controls and three RPL cases (34). In particular, a specific group of dNK cells expressing CSF1^+^ CD59^+^ killer immunoglobulin-like receptors (KIRs) was identified in normal deciduae. However, there was a reduction in the proportion of this particular subgroup in the RPL group (34). Additionally, their data indicated that during early pregnancy, decidual CD8^+^ T cells demonstrated cytotoxic characteristics in both healthy and RPL pregnancies (34). The decidual macrophages in RPL patients exhibited elevated expression levels of genes associated with both M1 and M2 characteristics, setting them apart from the conventional classification of M1/M2 (34). These findings provided insights into the diverse immune responses in the decidua and identified potential immune variations associated with RPL (34). Another study investigated the different distribution pattern of dNK cells in three RPL patients and three pregnant women without any complications using scRNA-seq (35). Compared to healthy pregnant women, significant disruptions were observed in the polarization process of dNK cells in RPL individuals (35). Simultaneously, a notable reduction was observed in the transcriptional activity of the extracellular matrix (ECM) among RPL patients (35). An additional single-cell study has found that RPL is associated with pro-senescent decidual activity in the luteal phase endometrium, suggesting that detection and treatment before pregnancy may reduce the occurrence of RPL (36).

Aside from examining decidual immune cells, a study conducted a detailed analysis of immune cells in peripheral blood (37). This study identified a reduction in the percentages of CD4^+^ naïve T cells, CD8^+^ naïve T cells, and CD4^+^ memory T cells in cases of RPL. Conversely, an increase in the proportions of CD8^+^ effector T cells and mucosa-associated invariant T (MAIT) cells was observed among females with RPL (37). Furthermore, the analysis of sequencing data revealed elevated levels of pro-inflammatory cytokines in both CD8^+^ effector T cells and MAIT cells, suggesting that these cells are in an abnormally activated state in RPL (37). Additionally, compared to collecting decidua, it is more convenient to collect peripheral blood at various time points, including before conception and during different gestational ages. Therefore, utilizing scRNA-seq information from immune cells in the periphery could potentially facilitate the early detection of abnormal pregnancy before symptoms manifest. It is crucial to emphasize that alterations in the immune system of any organ can influence the immune profile observed in peripheral blood. Thus, thorough verification and interpretation are necessary to determine if and how the immune status of peripheral blood accurately reflects the immune status of the maternal-fetal interface.

Recurrent abortions have been identified as being associated with an inflammatory condition and immune activation of the decidua, as indicated by various studies. Additionally, several molecular mechanisms linked to the occurrence of miscarriages have been revealed. These findings offer potential enhancements for preventing, identifying, and managing adverse pregnancy outcomes. Timely therapeutic intervention aimed at restoring the functionality of immune cells in decidual tissues could potentially contribute to preserving pregnancies. However, primary constraints for scRNA-seq investigations in RPL include challenges related to obtaining representative samples and addressing ethical concerns, both contributing to an elusive understanding of its pathogenesis. The mechanistic study of RPL could be significantly improved by having an ample number of samples from a diverse range of groups with varied backgrounds. Furthermore, differentiating between alterations in scRNA-seq that contribute to miscarriage and those resulting from abortion remains challenging. Additionally, practitioners often perform placental single-cell separation a few days after fetal death, potentially impacting the accuracy of experimental results due to the presence of fetal remnants in the uterus. Controls in these studies involve the elective termination of pregnancies without identified abnormalities. However, the use of these controls is based on the hypothesis that these pregnant women would have successfully completed their pregnancies, which is impossible to confirm.

## 5 PE

PE is a condition of unknown cause that occurs during pregnancy, with a prevalence ranging from 2% to 8%, typically manifesting after the completion of 20 weeks of gestation. This disease is characterized by elevated blood pressure, the presence of protein in the urine, and various impairments to vital organs including the brain, heart, liver, and kidneys (38). Patients may experience severe seizures that can lead to maternal death or fetal distress. Consequently, PE is one of the significant contributors to adverse pregnancy outcomes (39). Several findings have indicated that the primary factors contributing to PE are associated with the placental origin theory, specifically inadequate trophoblast invasion and impaired functionality of uterine spiral arteries resulting in shallow implantation of the placenta (40). The most effective approach to address PE is by delivering the fetus and the placenta, making it a prominent factor contributing to premature birth. Given the clinical and molecular heterogeneity of PE, the utilization of omics technologies such as transcriptomics, epigenomics, proteomics, and metabolomics could potentially enhance the understanding of the intricate molecular pathways underlying this disease (41–43).

Several studies have conducted human placental scRNA-seq analyses in PE (**Table 3**). The primary focus of these studies has been on investigating trophoblast cell populations and their transcriptomic profiles, given their significant role in the development of PE. One study examined transcriptomic changes specific to different cell types through unbiased scRNA-seq of placental samples, including two individuals with PE and two normotensive pregnant women (44). This comprehensive analysis involved 29,008 cells across 11 distinct cell types, encompassing trophoblasts and immune cells (44). The study explored the roles and characteristics of trophoblast subtypes in both the PE and control groups, identifying the potential involvement of CEBPB and GTF2B in the dysfunction of EVT in PE (44). Another study analyzed over 24,000 placental cells from both normal controls and preeclamptic mothers (10). Scientists integrated placental characteristic genes obtained from scRNA-seq with the analysis of circulating RNA in maternal plasma (10). This groundbreaking research demonstrated the potential for non-invasive methods to diagnose PE earlier by examining cellular changes during pregnancy. In a separate investigation, placentas were extracted following cesarean delivery (45). Researchers observed an elevation in gene expression related to endoplasmic reticulum signals in STB among individuals with PE (45). Additionally, the placental immune response in PE patients was found to be impaired. The newly identified VCT cell type exhibited heightened activity in proteasomes, spliceosomes, ribosomes, and mitochondria (45). More recently, researchers utilized scRNA-seq to identify genes expressed differently in the placentas and peripheral blood transcriptomes of patients with EOPE and late-onset PE (LOPE) (46). Experimental validation of blood samples confirmed the presence of EBI3, IGF2, ORMDL3, GATA2, and KIR2DL4 as novel biomarkers distinguishing EOPE from LOPE (46). This research reveals distinct pathogenic mechanisms of EOPE and LOPE and provides new targets for the early diagnosis of PE (46).

**Table 3:** Human placental scRNA-seq studies in preeclampsia (PE).

Reference	Gestational age (weeks)	Population	Samples	Number of cells	Single cell isolation	Platform	Average reads length (bp)	Mean pair-end reads	Data repository
Tsang (10)	Full-term, 24-33^+6^ weeks	Chinese	PE (n=8) *vs.* normal pregnancies (n=4)	24,000	Enzyme, centrifugation	10× Genomics	130	35,800	EBI: EGAS00001002449
Guo (46)	31-37 weeks	Chinese	EOPE (n=7) *vs.* LOPE (n=6)	N/A	N/A	Python (version 3.6.6)	N/A	N/A	ArrayExpress: E-MTAB-6701, FCA7196220, FCA7196226, FCA7474064, FCA7474065, FCA7474068, FCA7511884
Zhang (45)	34-38 weeks	Chinese	PE (n=3) *vs.* healthy pregnancies (n=3)	11,5181	N/A	Illumina HiSeq X	N/A	150	N/A
Zhou (44)	32-40 weeks	Chinese	Severe PE (n=2) *vs.* normal pregnancies (n=2)	29,008	Enzyme, centrifugation	10× Genomics	N/A	N/A	GEO: GSE173193
Yang (88)	LOPE: 34-37 weeks; normal pregnancy: more than 38 weeks	Chinese	Placenta: LOPE (n=3) *vs.* normal pregnancies (n=3); decidua: LOPE (n=3) *vs.* normal pregnancies (n=4)	N/A	Enzyme, centrifugation	Novaseq (Illumina, novaseq6000)	N/A	N/A	N/A
Luo (89)	Third trimester	Chinese	PE (n=2) *vs.* normal pregnancies (n=2)	101,250	N/A	BD Rhapsody and Illumina HiSeq 4000	N/A	N/A	GSA: HRA004699

EOPE, early-onset PE; LOPE, late-onset PE.

Consolidating these investigations underscores the impact of PE on various cellular signals and functions in both maternal and fetal cells at the maternal-fetal interface. This highlights the intricate pathogenesis of this disease and suggests the potential for using differentially expressed genes revealed by scRNA-seq analysis as biomarkers for PE. However, it should be noted that the number of research subjects in these studies is relatively small. Moreover, beyond trophoblast malfunction, abnormalities in decidual cells can also contribute to the development of PE. Therefore, it is crucial to employ scRNA-seq technologies to examine other cell types present at the maternal-fetal interface in women with PE and investigate its underlying mechanisms. Further research is necessary to explore the potential of using placental single-cell markers in maternal blood for the detection of PE. Additionally, severe cases of PE often result in premature delivery. Consequently, when comparing these cases, it is essential to include similarly premature but non-preeclamptic cases. This approach helps eliminate any potential confounding factors related to full-term gestation, which may introduce unrelated dimensions and lead to observed differences solely attributed to the effect of gestation rather than the pathology of PE. Currently, there is no unanimous agreement in the field regarding the ideal control for preterm delivery cases with PE. Readers are recommended to thoroughly evaluate scRNA-seq results, regardless of which control group was utilized.

## 6 Preterm birth

Preterm birth is defined as the termination of pregnancy before 37 weeks of gestation. Its incidence rate is 10.9%, representing a global health concern (47). Preterm birth stands as the primary factor contributing to mortality in children under 5 years old and is associated with a heightened risk of growth retardation, cerebral palsy, pulmonary dysplasia, and neurological disorders (48). Potential factors contributing to preterm birth include maternal infections, polyhydramnios, multiple pregnancies, as well as complications such as hypertensive disorders of pregnancy and GDM (49).

In 2019, a comparative study was conducted to establish distinct gene signatures for specific cell types during both normal full-term pregnancy (38-40 weeks) and premature birth (33-35 weeks) (50). Tissues from placental villi, basal plate, and chorioamniotic membranes were collected for analysis. By analyzing the different expression levels of marker genes, a novel set of non-proliferative interstitial VCTs was revealed in both placental villi and the neighboring basal plate. A fresh group of decidual cells in the lymphatic endothelium was identified within the chorioamniotic membranes using gene signatures specific to this cell type. Gene enrichment analysis conducted with clusterProfiler suggested that decidual cells in the lymphatic endothelium have the potential to facilitate the migration of immune cells towards the chorioamniotic membranes. Furthermore, notable variances were observed in the transcriptional profiles of placental cell types between full-term pregnancy and preterm birth. Maternal macrophages from the chorioamniotic membranes exhibited the highest number of genes with differential expressions when comparing the term labor group to the group without labor at term. Additionally, computational analysis using publicly available datasets revealed that the circulating profiles of macrophages, monocytes, activated T cells, and fibroblasts exhibit alterations in women experiencing preterm labor compared to controls matched for gestational age. Further research is required to define the transcriptional patterns associated with extremely premature labor (less than 32 weeks).

## 7 GDM

The global prevalence of GDM is increasing. Relevant research suggests that offspring may experience adverse health outcomes as a result of exposure to elevated maternal blood sugar levels during pregnancy (51). Published reports imply that placentas from pregnancies affected by GDM consistently exhibit dysregulation in immune and inflammatory genes (51–54). Insulin resistance (IR) is considered as the key factor in the progression of GDM. Some findings reveal that the impact of GDM on IR varies across different tissues, and the placenta of women with GDM also shows compromised glucose tolerance and insulin sensitivity (55). Moreover, there is a growing body of evidence suggesting that placental abnormalities may have significant implications in the development of GDM (56).

One study was conducted to perform scRNA-seq analysis on GDM patients (57). Full-term placental samples were obtained from cesarean deliveries, including two normal pregnant women and two GDM cases. These specimens underwent scRNA-seq, resulting in a total of 14,591 GDM cells and 12,629 control cells for comparative analysis. Nine different types of cells were observed, including VCTs, STBs, EVTs, granulocytes, myelocytes, T/NK cells, B cells, monocytes, and macrophages. During subset analyses, pathways related to estrogen and antigen presentation were found to be enriched in trophoblasts, while IL-17 signaling showed a decrease in GDM. Furthermore, immune cell subgroups were identified and their correlation with GDM was examined, followed by confirmation using flow cytometry. In GDM patients, notable increases were observed in the numbers of NK cells and cytotoxic T cells, along with an enhancement of M2 (CD206^+^) macrophages. Additionally, there was a general reduction in the inflammatory response associated with GDM, as indicated by the absence of predicted ligand-receptor interactions involving RPS19-C5AR1, SPP1-PTGER1, and SPP1-CD44 complexes. A recent study based on scRNA-seq revealed that gestational diabetes subtype A1 (GDMA1) (controlled by diet and exercise), GDMA2 (requiring drug treatment) and pregnant women with type 2 diabetes had different genetic characteristics in placental cells (58). For example, genes involved in chromatin remodeling and epigenetic regulation were elevated in the placentas of GDMA1 and pregnant women with type 2 diabetes (58). However, there were more genes involved in wound healing pathway in the placentas of GDMA2 patients (58). These discoveries contribute to a better understanding of the molecular processes involved in GDM, potentially paving the way for novel strategies in its management and prevention.

## 8 Coronavirus disease 2019

The global outbreak of COVID-19, caused by the severe acute respiratory syndrome coronavirus 2 (SARS-CoV-2), has impacted over 10 million individuals, including expectant mothers. As of now, there is a lack of conclusive evidence supporting the vertical transmission of SARS-CoV-2. The virus typically utilizes the angiotensin-converting enzyme 2 (ACE2) receptor and TMPRSS2 serine protease to gain entry into cells. Recent findings indicate that a specific group of STB cells in early pregnancy and EVTs in the second trimester of the human placenta express ACE2 and TMPRSS2, as evidenced by scRNA-seq results in a public database (59). It is essential to note that the BSG/CD147 receptor, acting as an alternative entry point for SARS-CoV-2, is present in all placental cells (59). This observation suggests the possibility of multiple mechanisms facilitating viral penetration into these cells. Additionally, ACE2, DPP4, and ANPEP are expressed in the placenta along with the viral S protein proteases (59). However, ACE2 and TMPRSS2 were not detected at significant levels in single-nucleus RNA sequencing (snRNA-seq) data obtained from 26,501 cells collected from 32 placental villi and deciduae in the second and third trimesters (60). Despite this, instances of SARS-CoV-2 transmission from mother to child have been documented (61–63), and recent research has demonstrated that cultured placental cells exhibit low replication efficiency for SARS-CoV-2 (64). Consequently, further *in vivo* investigations are necessary to establish the factors contributing to the transmission or non-transmission of SARS-CoV-2 to the fetus. These publications emphasize the limitations associated with inferring biological processes solely based on individual transcript expression within extensive and unchanging datasets.

## 9 Placental sexual dimorphism

Biological gender significantly influences the immune regulatory network. In a study, researchers refrained from sorting or lysing red blood cells, and instead examined placental villi from six pregnant women (3 male and 3 female fetuses) from 6 to 11 gestational weeks (65). This approach resulted in a collection of 7,245 scRNA-seq transcriptomes with high quality. Cluster analysis effectively distinguished various cell types within the placental villi, including trophoblasts, stromal fibroblasts, Hofbauer cells, antigen-presenting cells, and endothelial cells, using important indicators. Subsequently, cells were categorized as either male or female to identify transcriptional alterations specific to each cell type. Noteworthy disparities were observed across different cell types, with males exhibiting elevated expressions of RPS4Y1, EIF1AY, and DDX37, while females displayed higher levels of MAGEA4, TMSB4X, and XIST. Upon reclassifying trophoblast cells, the emergence of seven distinct subtypes characterized by pseudotime trajectories diverging towards either HLA-G or ERVFRD-1 cell fates was observed, representing EVTs or STBs, respectively. Immunohistochemistry further identified the presence of MUC15 in STBs and NOTUM in EVTs. Notably, these genes exhibited significant sexual dimorphism and were transcribed from non-sex chromosomes. Relevant analyses indicated that TGFB1 and estradiol were identified as potential upstream mediators affecting each cell type, with a notable increase in the expression levels observed in males (65).

## 10 Peripartum study

A research conducted scRNA-seq analysis on the transcriptomes of peripartum decidua, examining 29,231 decidual cells both before and after childbirth (66). Various cell types, including endothelial cells and fibroblasts, as well as subtypes of decidual stromal cells, EVTs, and T cells, were detected and observed to possess diverse functionalities. In comparison to the pre-delivery period, there was a noticeable activation of decidual stromal cells, EVTs, and various T-cell subtypes following delivery. This activation included an increase in cell growth and the stimulation of various cell signals, such as the activator protein 1 pathway. The findings from pseudotemporal ordering revealed heterogeneity in the processes of decidualization (decidual stromal cell) and infiltration (EVT) between prenatal and postpartum periods (66).

## 11 Polycystic ovary syndrome

PCOS is a prevalent condition among women of reproductive age, affecting up to 15% of the global population (67). It is defined by a range of clinical manifestations, such as inability to conceive, excessive androgen levels, irregular periods, being overweight, and resistance to insulin (68–71). Despite thorough investigation, there is still a lack of complete comprehension about the molecular mechanisms involved in the etiology of PCOS. One study utilized scRNA-seq to compare the gene expression differences in passaged cultures of theca cells derived from ovaries of 5 women with normal ovulation and 5 PCOS patients (72). Through the application of bulk RNA-seq and microarray studies, it was verified that the expression profiles are primarily influenced by elevated expression or activity of the transcription factor SREBF1, which controls genes involved in cholesterol acquisition (LDLR, LIPA, NPC1, CYP11A1, FDX1, and FDXR), and GATA6, which orchestrates the level of genes encoding the steroidogenic enzyme (CYP17A1) in conjunction with other diversely expressed transcription factors (SP1, NR5A2) (72). The present research offers information about the molecular processes involved in the hyperandrogenemia linked to PCOS and identifies possible research directions for molecular diagnosis and treatment.

A different research project examined the data from scRNA-seq in the previous study, which included information on 14 oocytes from 7 fertile women and 20 oocytes from 9 females with PCOS at the germinal vesicle (GV) phase, metaphase I (MI) phase, and metaphase II (MII) phase (73). The GV phase showed notable enrichment of genes associated with mitochondrial regulation, as confirmed through functional enrichment analysis and gene co-expression network analysis (73). The results of principal component analysis (PCA) and differential gene expression analysis indicated a significant difference in GV compared to the MI and MII phases between the two groups (73). Further examination revealed that the increased expression of genes at the GV phase in individuals with PCOS is primarily associated with mitochondrial function, including COX6B1, COX8A, COX4l1, and NDUFB9 (73). In healthy cells, these genes were found to be stimulated at the MII phase, while in PCOS oocytes, it appears that some mitochondrial functions are stimulated at the GV phase in advance (73). In short, this study demonstrated that abnormal mitochondrial activity during the GV phase could be a factor in the deterioration of oocyte quality in PCOS cases.

In a similar manner, another group used scRNA-seq data from human adult ovaries (74) to assess the function of ovarian cells in PCOS through integrating different transcriptomic information from granulosa cells (GCs) (75). Twenty-two unique cell clusters of human ovarian cells were effectively identified. Subsequently, a gene expression matrix containing 13,904 genes across 30 samples (15 normal *vs.* 15 PCOS) was obtained through transcriptome integration (75). After further deconvolution analysis, they found that there was a reduction in the percentage of small antral GCs and an increase in the percentage of KRT8^high^ mural GCs, as well as HTRA1^high^ cumulus cells in PCOS. This was particularly evident in the elevated transition from small antral GCs to KRT8^high^ mural GCs (75). Furthermore, a team of researchers conducted initial screening, categorization, functional assessment, and identification of target genes and potential drugs using scRNA-seq data obtained from 34 oocytes in the GEO database (76). It was discovered that oxidative phosphorylation had a significant impact on the maturation of oocytes, as well as insulin sensitivity and disorders related to ovulation (76). PGR, SIRT1 and ADAMTS1 were identified as central differentially expressed genes (DEGs) and potential drugs were discovered through the Drug-Gene Interaction Database (76). These three genes may contribute to the development of drugs for treating PCOS. Additionally, a separate scRNA-seq analysis demonstrated that PCOS patients exhibited decreased expression of genes related to meiosis, including EGFR, PGR, PGRMC1, PLCZ1, SFRP4, ZMIZ1, and ZSCAN4. Conversely, the expression of genes associated with DNA repair such as XRCC1, LIG, and RAD54L was found to be elevated in PCOS individuals (77). Collectively, these studies offer knowledge of the molecular changes and cell makeup in the ovarian tissues of PCOS, which may aid in comprehending the pathophysiology of PCOS and provide a valuable resource for basic research on this condition.

## 12 Knowledge gaps and prospective outlooks

The placenta is a multicellular organ derived from both the mother and the fetus. The scRNA-seq investigations conducted on human placentas demonstrate significant variations in gene expression across different regions of the placenta, among diverse cell populations, at various stages of pregnancy, and in cases of pregnancy-related disorders. These findings provide opportunities for an enhanced comprehension of cellular networks and pathways in both typical and intricate pregnancies, potentially leading to the identification of fundamental factors contributing to unfavorable pregnancy outcomes. However, attention should be paid when using the human placenta for scRNA-seq analysis. Firstly, accessing a healthy human placenta prior to delivery poses ethical challenges, restricting the scope of research that can be carried out. Secondly, during delivering, it is important to consider multiple factors such as hospital protocols, delivery methods, and the sizes of tissues. Moreover, the placenta undergoes growth and continuous adaptation in response to the maternal environment, aiming to maintain a healthy pregnancy. Consequently, it is inaccurate to hold fixed perspectives on the proportions of placental cells or gene expression.

Additionally, scRNA-seq necessitates the use of freshly obtained samples and a substantial proportion of viable cells following dissociation, posing challenges in specimen acquisition and increasing variability across experimental batches (78). To address these issues, an alternative approach, called snRNA-seq, has emerged as a viable option to traditional single-cell techniques. Requiring no enzymatic digestion, snRNA-seq minimizes the occurrence of dissociation artifacts. Furthermore, snRNA-seq offers the advantage of being applicable to frozen tissues, eliminating the need for the preparation of live single cell suspension from fresh tissues. This eliminates any potential batch effects and allows for simultaneous single-cell gene profiling of archived clinical samples. Consequently, this approach enhances the study’s statistical power by enabling an increased number of biological replicates (79).

## 13 Conclusion

Active investigation into pregnancy-related diseases utilizing scRNA-seq techniques has led to the swift accumulation of novel discoveries. scRNA-seq may stand out as one of the crucial tools for studying the etiology of pregnancy complications. In comparison to bulk RNA-seq, scRNA-seq technology offers the advantages of identifying cellular diversity and uncovering concealed variations in gene expression. It also allows for the investigation of intercellular communications within tissues. This, in turn, facilitates faster and more precise detection and surveillance of different courses of pregnancy complications. Currently, integrating the scRNA-seq technique with bulk RNA-seq analysis allows for significant discoveries while mitigating the constraints associated with each method. In the foreseeable future, an increase in research endeavors aimed at uncovering the typical progression of placenta development during a normal pregnancy is anticipated. These studies may contribute to the development of a comprehensive dynamic atlas of the placenta, akin to those found in the Pediatric Cell Atlas (PCA) (80), despite facing various technological and practical obstacles. Additionally, different lifestyles can have a significant impact on pregnancy, such as smoking or drinking during pregnancy. The scRNA-seq studies can also be conducted on this population to analyze the biological changes occurring in the placenta. Furthermore, the advancement of single cell technologies opens up opportunities to explore other types of single cell omics investigations, such as studying epigenetic modifications at the single cell level. Conducting multi-omics analyses on individual cells using techniques like scRNA-seq, sc-methylation, and scATAC-seq can help understand the interconnected changes occurring from epigenetics to transcriptomics (81, 82). In addition to pipelines and workflows for scRNA-seq, incorporating tools and techniques that focus on meta-analysis or comparative research of diverse datasets would be beneficial for revealing the developmental trajectory of the placenta across different stages of gestation. There is a significant demand for methodologies that combine various types of data from individual cells and multiple modes, especially those capable of analyzing spatial transcriptomics data, to achieve a more comprehensive understanding of placental tissues. It will be intriguing to observe whether and how the techniques for integrating multi-omics data in bulk cell analysis can be expanded to single-cell analysis (83). Furthermore, the objective of single-cell investigations is not to replace previous bulk RNA-seq studies, which have extensive sample sizes and significant statistical strength. Instead, their purpose is to enhance these studies by incorporating cell type specificity (84). Looking ahead, the future direction of scRNA-seq applications will involve devolving into functional biology, with a primary focus on understanding variations in transcriptional activity among highly specific cell populations.
